# Ex vivo evaluation of intravitreal mesenchymal stromal cell viability using bioluminescence imaging

**DOI:** 10.1186/s13287-018-0909-y

**Published:** 2018-06-13

**Authors:** Carolina Assis P. Vilela, Lucas Eduardo B. Souza, Rubens C. Siqueira, Rodrigo T. Calado, Dimas T. Covas, Jayter S. Paula

**Affiliations:** 1 Department of Ophthalmology, Otorhinolaryngology and Head and Neck Surgery, Ribeirão Preto Medical School, University of São Paulo, Av. Bandeirantes, 3900 – 12. Andar, Ribeirão Preto, São Paulo 14049-900 Brazil; 2 Hemotherapy Center of Ribeirão Preto, Ribeirão Preto Medical School, University of São Paulo Ribeirão Preto, São Paulo, Brazil

**Keywords:** Bioluminescence, Cell transplantation, Cell viability assessment, Intravitreal injection, Luciferase, Mesenchymal stromal cell

## Abstract

**Background:**

Bone marrow-derived mesenchymal stromal cell (MSC) therapy is a promising treatment for several degenerative ocular diseases; however, no reproducible method of monitoring these cells into the eye has been established. The aim of this study was to describe successful bioluminescence imaging (BLI) to detect viable luciferase-expressing MSC in the eye.

**Methods:**

Human donor MSC in culture were transduced with 50 μl luciferase lentiviral vector (three viral particles/cell) prior to intraocular injection. Twenty-one right eyes of 21 rabbits were evaluated through BLI after receiving 1 × 10^6^ luciferase-expressing MSC intravitreally. Contralateral eyes were injected with vehicle (phosphate-buffered saline (PBS)) and were used as controls. At seven different time points (1 h to 60 days), d-luciferin (40 mg/ml, 300 μl PBS) was injected in subsets of six enucleated eyes for evaluation of radiance decay through BLI analysis. CD90 and CD73 immunofluorescence was studied in selected eyes.

**Results:**

Eyes injected with MSC showed high BLI radiance immediately after d-luciferin injection and progressive decay until 60 days. Mean BLI radiance measures from eyes with luciferase-expressing MSC were significantly higher than controls from 8 h to 30 days. At the thirtieth day, positive CD90- and CD73-expressing cells were observed only in the vitreous cavity of eyes injected with MSC.

**Conclusions:**

Viable MSC were identified in the vitreous cavity 1 month after a single injection. Our results confirmed BLI as a useful and reliable method to detect MSC injected into the eye globe.

## Background

Mesenchymal stromal cells (MSC) are considered as a specific multipotent population of cells that can be isolated from some tissues including bone marrow. These cells are different to hematopoietic stem cells mainly because they are adherent to dishes and exhibit fibroblast-like colonies in culture, besides being positive for several specific cellular markers [1, 2]. The use of MSC was initially based on their potential differentiation into several tissues; however, an immunomodulatory property has been recently proposed [3].

These findings support the rationale of developing strategies for MSC transplantation as a regenerative therapy, especially in highly specialized organs [2–6]. The in vivo function of MSC probably depends on the pathologic stimuli that each tissue has experienced. In this context, ocular tissues may represent a good target for studying MSC actions since several neuroretinal diseases have different degrees of underlying inflammation associated with a progressive lack of highly specialized cells [7, 8].

The major problem related to the use of MSC in the eye, as well as other organs, is the difficulty in assessing cellular survival and activity continuously, which are key points for determining the efficacy of treatment and the best MSC transplantation protocols [9–14]. In vivo optical bioluminescence imaging (BLI) is a newly developing technology for dynamically observing the biological behavior of viable cells. BLI allows the detection of light emitted by cells expressing light-generating enzymes [15]. Using this technology, the cells of interest are genetically modified to express luciferase, an enzyme that generates visible light through the oxidation of specific substrates such as d-luciferin [16]. In this study, we demonstrate the feasibility of using BLI to detect intraocular viable luciferase-expressing MSC in an ex vivo rabbit eye model, confirmed by immunofluorescence assays.

## Methods

### Isolation of human MSC

Bone marrow tissues of normal subjects were harvested for allogenic bone marrow transplantation after informed consent according to routine institutional protocols. In the present study, mononuclear cells from a single donor were centrifuged over a Ficoll-Hypaque gradient (Sigma, St. Louis, MO) and plated in 180 cm^2^ dishes with 10 ml MSC in alpha-minimum essential medium (MEM; Gibco Life Technologies, Burlington, ON, Canada) plus 15% fetal bovine serum (FBS). The nonadherent cells were removed after 3 days by washing with phosphate-buffered saline (PBS), and monolayers of adherent cells were cultured until confluence. Then, cells were trypsinized (0.025% trypsin/0.1% EDTA/sodium pyruvate) and plated at 5000 cells/mm^2^ density for use at passages 3 to 4 for the experiments.

Passage three MSC were tested with various differentiation media according to the manufacturer’s recommendations (Miltenyi Biotec, Auburn, CA) to determine their potential for differentiation. Cellular phenotyping was performed for positivity for CD73, CD90, CD105, and negativity for CD14, CD34, CD45, and HLA-DR expression according to the International Society for Cellular Therapy [17, 18].

### MSC transduction

A recombinant lentiviral vector expressing luciferase-2 (Luc2) was incubated with MSC using a three viral particles/cell proportion. Detection and viability of transduced MSC were made using quantitative real-time polymerase chain reaction (qRT-PCR). Detailed steps of the protocol used are described by Kidd et al. [19] and modified locally by Meirelles (unpublished data). In summary, our lentiviral vector is bicistronic and codes for luciferase-2 and puromycin-N-acetyltransferase, which confers cellular resistance to puromycin. We performed transduction of MSC at the first passage using the mentioned multiplicity of infection (3:1). After 48 h of transduction, cells were incubated in 2 μg/ml puromycin for 6 days. This treatment leads to 100% of death in nontransduced MSC (i.e., 100% of transduced MSC in culture). After puromycin selection, all the isolated transduced MSC were expanded and transplanted at the third passage, as explained previously. The transduced MSC used in this study were kindly provided by the Hemotherapy Center of Ribeirão Preto, Ribeirão Preto Medical School, University of São Paulo.

### Ex vivo model of intravitreal injection of MSC

A preliminary study was performed using three eyes of two New Zealand rabbits (*Oryctolagus cuniculus*) euthanized with intravenous pentobarbital (150 mg/kg).

Two eyes (E1 and E2) received an intravitreal single dose of 1 × 10^6^ transduced MSC (300 μl) through a pars plana injection using a 30-Gauge needle. To evaluate immediate (using E1) and delayed (using E2) intraocular viability of the transduced MSC, cells were suspended in 300 μl PBS with (E1) or without (E2) d-luciferin (40 mg/ml). A third eye (E3) received a pars plana intraocular injection of d-luciferin (40 mg/ml in PBS, 300 μl) without cells. At 1 h after the intraocular infusions, E2 received an additional intravitreal injection of d-luciferin (40 mg/ml in PBS, 300 μl) without cells. All eyes underwent a protocol for bioluminescent detection from time zero for 8 h as further described. This set of experiments was repeated twice and was used to observe how long the MSC would produce luminescence considering the dose of d-luciferin used.

After analyzing the preliminary results, the study was extended until 8 weeks. A one-time intravitreal injection of transduced MSC was then performed in live rabbits to observe the bioluminescent decay curve. Eighteen right eyes of 18 animals received a single 30-Gauge pars plana intravitreal injection of 1 × 10^6^ transduced MSC (300 μl) at time point zero. Left eyes received a pars plana injection of 300 μl vehicle and served as controls.

At each time point, three animals were euthanized using intravenous pentobarbital (150 mg/kg) and the eyes were enucleated. Thus, each eye received a pars plana intravitreal injection of d-luciferin (40 mg/ml in PBS, 300 μl) and was used immediately for bioluminescent detection as further described.

All the animals were donated by the Central Bioterium of the University of São Paulo (Ribeirão Preto Campus). The animal procedures were performed in accordance with the ARVO Statement for the Use of Animals in Ophthalmic and Vision Research. We also obtained approval from the Ethics Committee on Animal Experiments of the School of Medicine of Ribeirão Preto, University of São Paulo.

### Bioluminescent detection of transduced MSC

Bioluminescent images were obtained using the IVIS Lumina Imaging System® (Caliper LifeSciences). The authors set the exposure time at 1 s and the luminescent signal was quantified in the images through the Living Image® Version 3.0.4 software (Caliper LifeSciences). The obtained signals were presented in photons/seconds (p/s) in the interest region (cm^2^) in 6-min cycles. For the comparative analyses between the time points, peak values of the luminescent signal results were considered.

### Immunofluorescence assays

At the 30- and 60-day time points, after bioluminescent analyses, all eyes were prepared for routine histology (hematoxylin and eosin (H&E)) and immunostaining for human CD90 and CD73. Whole globes were fixed in 10% buffered formalin, embedded in paraffin, and transferred to microscope slides after 6-μm anteroposterior microsectioning (Leica Jung RM2065, Leica Microsystems GmbH, Wetzlar, Germany) and mounted using Tissue Tek® Glas™ Mounting Media (Sakura Finetek USA).

The immunostaining assays were performed after deparaffinization and rehydration with xylene and descending ethanol series. Then, slices were incubated with the primary antibody anti-CD90 (#555593, BD Biosciences Pharmingen) and anti-CD73 (# 550256, BD Biosciences Pharmingen) at 1:200 in 1% bovine serum albumin (BSA) solution for 2 h followed by three-times PBS washing, and incubation with the secondary antibody Alexa Fluor® 647 (BioLegend, San Diego, CA, USA) at 1:500 for 1 h at room temperature in darkness. The slides were mounted with ProLong® Gold Antifade Reagent with DAPI (Life Technologies, Carlsbad, CA, USA). The images were obtained using a digital microscope (Leica Microsystems, DFC 310 FX, Wetzlar, Germany). Images of retinal and intravitreal histology and cell immunostaining were compared between eyes that underwent transduced MSC injections and controls. In conducting immunostaining assays we considered the MSC lack of immunogenicity at different sites in addition to the vitreous cavity characteristics of being in an immune-privileged host environment.

### Statistical analysis

Data are presented through descriptive statistics and scattered plot graphs. Comparisons of Log-transformed results of luminescent signals were made using *t* tests (Prism 6.0, GraphPad software Inc., CA, USA).

## Results

**Fig. 1:**
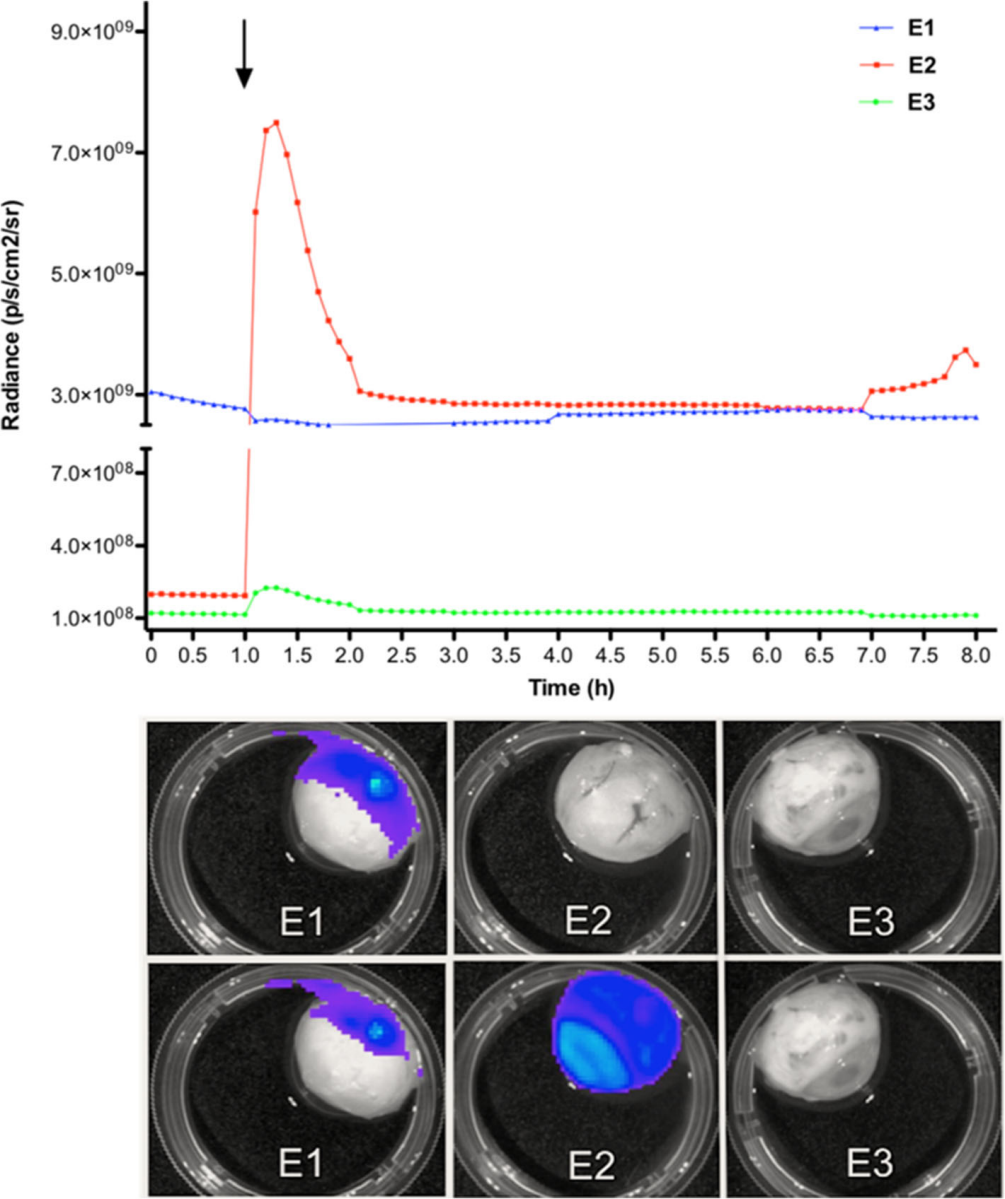
Bioluminescence results obtained from the three experimental eyes in the preliminary study. Top panel shows the distribution of the total amount of captured photons/second from the three eyes during the 8-h period. Bioluminescent levels were obtained from E1 (MSC + d-luciferin (40 mg/ml, 300 μl), E2 (MSC before and after injection of d-luciferin (40 mg/ml, 300 μl); black arrow), and E3 (d-luciferin (40 mg/ml, 300 μl)). Bottom panel shows bioluminescence imaging acquired at *t* = 0 h (first row) and *t* = 1.5 h (second row) after the additional injection of d-luciferin (40 mg/ml, 300 μl) performed in E2

**Fig. 2:**
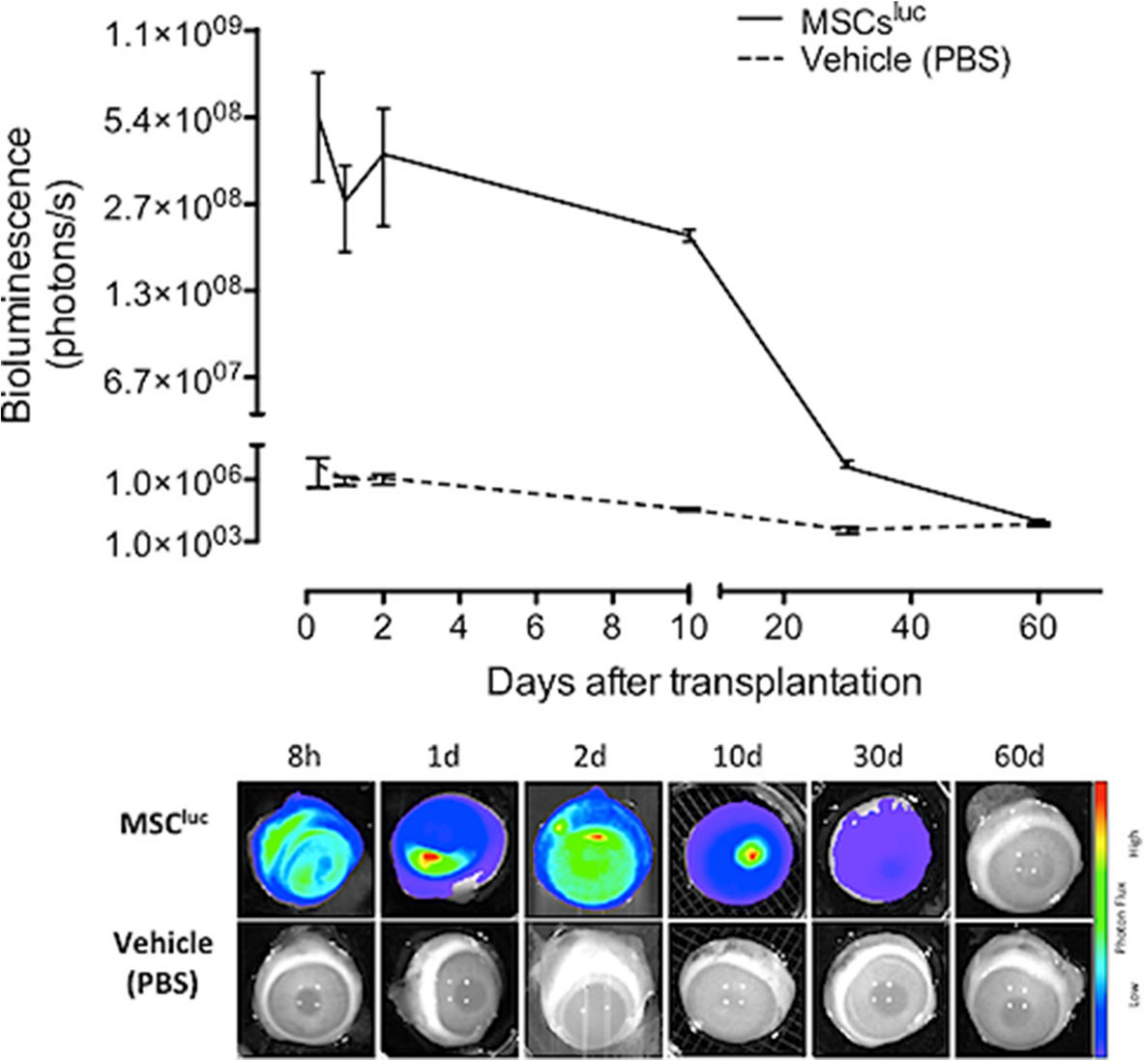
Bioluminescence results during the 60 days of follow-up. Top panel shows the distribution of the total amount of captured photons/second at six time points during the 60 days after the intravitreal injection of transduced mesenchymal stromal cells (MSC) in rabbit eyes (solid line) compared with controls (phosphate-buffered saline (PBS); dashed line). Mean ± standard error (error bars) of bioluminescent levels were obtained from triplicates of eyes at each time point (methodological details are presented in the main text). All readings (from 8 h to 30 days), apart from those at day 60, were significantly higher than controls. Bottom panel shows examples of bioluminescence imaging acquired from eyes injected with transduced MSC (MSC^luc^; first row) or controls (vehicle/PBS; second row)

Immediately postinjection, an intraocular luminescent signal from transduced MSC was detected only in E1 (Fig. 1). A Kruskal-Wallis test showed a significant difference between E1, E2, and E3 at the beginning (*p* < 0.001; mean ± standard deviation for E1, E2, and E3 was 2.91 × 10^9^ ± 9.0 × 10^7^, 1.97 × 10^8^ ± 2.37 × 10^6^, and 1.18 × 10^8^ ± 1.94 × 10^6^, respectively; Fig. 2). After intravitreal injection of d-luciferin in E2, an immediate peak of luminescence was observed (7.50 × 10^9^), with decay to the E1 levels over a 1.5-h period. E2 maintained the plateau levels of E1 luminescent until 8.0 h, with no observed changes in the background levels of E3 (Figs. 1 and 2).

A significant decreasing detection of the luminescent signal was observed at 8 h and 1, 2, 10 and 30 days after the intravitreal injection of transduced MSC (*p* < 0.05). At 60 days after injection, the luminescent levels were similar to the control eyes (Fig. 2).

**Fig. 3:**
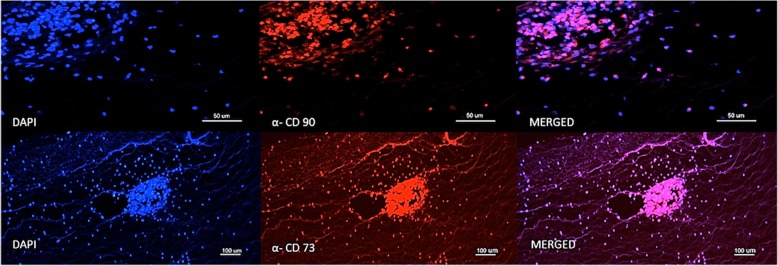
Immunofluorescence expression of cells located into the vitreous cavity of eyes harvested at day 30 after the intravitreal injection of transduced MSC. Top panels: anti-CD90 counterstained with DAPI; bottom panels: anti-CD73 counterstained with DAPI

**Fig. 4:**
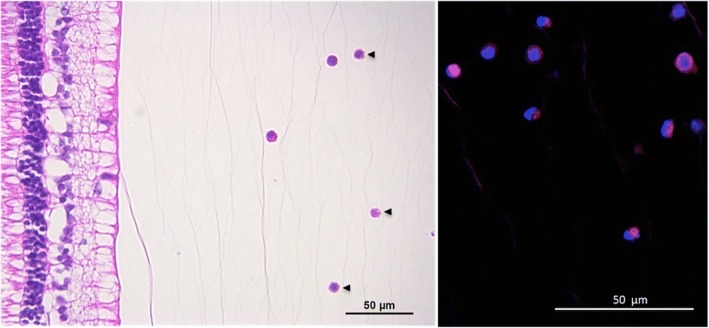
Photomicrography (left) and immunofluorescence images of cells (right) taken 30 days after the transplantation of the transduced MSC. Note few cells located close, but not attached, to the retina layers (right, H&E staining; magnification 400×). Some shrinking cells may be observed (arrowheads) indicating cell death at that time. At a higher magnification, the immunofluorescence expression of anti-CD90 could be verified on the surface of some cells in selected microscopic fields of the vitreous cavity (anti-CD90, counterstained with DAPI; magnification 630×)

Histological evaluation at day 30 showed cells located exclusively in the vitreous cavity of eyes injected with transduced MSC, which could be described as lymphomononuclear cells based on their morphological characteristics. No cells were found in any other ocular structures. Virtually all cells displayed in the vitreous cavity were both CD73 and CD90 positive (Figs. 3 and 4), and no cells were detected in the vitreous cavity of all eyes at day 60.

## Discussion

Stem cell-based therapies have rapidly emerged as potential therapeutic approaches for several pathologic conditions. Their multiplicity of potential action has been evaluated in several diseases since MSC may present various properties, including transdifferentiation into several cells types and modulation of the microenvironment [20–24]. In fact, their paracrine effect is potentially related to the modulation of the activity of immune cells, including inhibition of B cell and natural killer cell proliferation and neutrophil activation, which would be mediated by several secreted factors [25–28]. Although these effects could be beneficial for different eye conditions, including retinal vascular disease and glaucoma [29–33], a recent study has reported severe ocular complications using intravitreal adipose tissue-derived MSC to treat patients with age-related macular degeneration [34].

Reliable markers to track MSC viability in vivo are needed, mainly in terms of cell tracking in the organs where cells are transplanted, especially into the eye. Several recent studies have reported the use of stem cells to treat ocular pathologic conditions [35–39].

Unlike end-point studies, BLI provides real-time, noninvasive assessment of in situ events, giving a better understanding of the kinetics of a biological process at multiple time points with the use of less animals. In this study, we demonstrated the viability of luciferase-expressing MSC injected intravitreally in a significant amount up to 30 days after injection. This is a new interesting advance regarding monitoring living cells. Previous reports have shown their beneficial effects in several ocular conditions; however, they have failed to show the maintenance of living transplanted cells in the eye [40]. Shi et al. [41] showed the presence of endothelial progenitor cells labeled with several concentrations of 5- (and 6)-carboxyfluorescein diacetate succinimidyl ester (CFSE) in laser-injured mouse retina using angiography. Similar to our results, they described decreases in the fluorescence intensity at the end of 4 weeks. Notwithstanding, BLI has been used for imaging MSC injected into the myocardium, kidney, and subcutaneous scaffolds [42–46]. This widespread use of BLI shows its reproducibility as a method for tracking injected MSC, and highlights the importance of testing BLI protocols for a time-course evaluation of intraocular MSC.

In the preliminary tests, as expected, d-luciferin applied together or after luciferase-expressing MSC provided reliable luminescent observations, reaching the same ranges. Nevertheless, we observed a rapid peak of luminescent when d-luciferin was injected after transfected cells have been injected (E2). The luminescence reached higher levels than those presented when d-luciferin and cells were injected together (E1). The explanation for this observation could be based on the increased enzymatic activity of cells in E2 before receiving d-luciferin, which would lead to higher photon flux; however, this explanation is open to debate.

The luminescent signal decay during the experimental time points (8 h until 60 days) could be attributed to losses of viable MSC. Shi et al. [41] have also reported the decrease in fluorescence intensity during the extended culture period as well as in an in vivo model.

The clinical interest regarding safety and efficacy of using human MSC, as well as ethical limitations and its potential further applications for treating eye disorders, justifies our proposal for the xenograft ex vivo rabbit model. Furthermore, based on the lack of signs of any ocular inflammation, such as vitritis or retinitis, the present model may be used in future studies on stem cell treatment of eye diseases since MSC usually present with low immunogenicity, and the vitreous cavity may work as an immune-privileged host environment. Besides, the immunofluorescence assays were performed to confirm our bioluminescence results regarding the presence of MSC into the vitreous cavity. At day 30, virtually all intravitreal cells retained their immunophenotype because of both human CD73 and CD90 positivity, indicating these as the injected MSC. Comparing the global bioluminescence levels between the time points of 8 h and 30 days, approximately 0.6% of the transduced MSC would be alive in the vitreous cavity at day 30. Although MSC could be taken up by phagocytes, we believe that intact enzymes of live cells left over (as observed in Figs. 3 and 4) were able to produce the source of light detected. We did not perform a histological estimation of the number of cells at that time because of the likely biased serial counting associated with potential loss of vitreous material during the preparation of the slices.

Summarizing, the immunofluorescence positivity to human CD73 and CD90 and the observed residual activity of the luciferase indicate a long-lasting, low immunological response to the transduced human MSC in this xenogeneic model. We speculate that either the previously described “immune privilege” of the vitreous or the local immunosuppressive effects of the injected MSC would explain our results. Although our results are straightforward, the use of human MSC from a single donor could be considered a limitation due to a potential heterogeneity of the cell biology.

Thus, the present ex vivo model of rabbit eyes used to evaluate MSC location and viability is a valuable additional tool for monitoring transplanted cells to the eye. Nonetheless, its clinical application in humans has not yet been studied and requires further safety trials. The possibility of repeated d-luciferin injections in the eyes of the rabbits represents an attractive advantage; however, additional evaluations of this method and a more in-depth understanding on phagocytosis processes are necessary to determine long-term dynamics of viable MSC and their mechanisms of action.

## Conclusions

Our ex vivo model using rabbit eyes demonstrated that transplanted viable MSC could be detected in the vitreous cavity 1 month after a single eye injection. These results may confirm BLI as a useful and attractive method to detect modified MSC injected into the eye globe.

## Abbreviations

BLI: Bioluminescence imaging
MSC: Mesenchymal stromal cells
